# Health worker perspectives on the possible use of intramuscular artesunate for the treatment of severe malaria at lower-level health facilities in settings with poor access to referral facilities in Nigeria: a qualitative study

**DOI:** 10.1186/s12913-016-1811-x

**Published:** 2016-10-12

**Authors:** Olatunde Adesoro, Constance Shumba, John Kpamor, Jane Achan, Harriet Kivumbi, John Dada, Kolawole Maxwell, James Tibenderana, Madeline Marasciulo, Prudence Hamade, Olusola Oresanya, Joanita Nankabirwa, Ebenezer Baba

**Affiliations:** 1 Malaria Consortium Nigeria, 3rd Floor, Abia House, Off Ahmadu Bello Way, Central Business District, Abuja, F.C.T. Nigeria; 2 Malaria Consortium Africa Region, P.O Box 8045, Kampala, Uganda; 3 Malaria Consortium, Development House, 56-64 Leonard Street, London, EC2A 4LT UK; 4 Medical Research Council Unit The Gambia, P.O Box 273, Serrekunda, The Gambia

**Keywords:** Intramuscular artesunate, Severe malaria, Lower-level facilities, Poor referral systems, Qualitative study

## Abstract

**Background:**

Innovative strategies are needed to reduce malaria mortality in high burden countries like Nigeria. Given that one of the important reasons for this high malaria mortality is delay in receiving effective treatment, improved access to such treatment is critical. Intramuscular artesunate could be used at lower-level facilities given its proven efficacy, ease of use and excellent safety profile. The objective of this study was therefore to explore health workers’ perspectives on the possible use of intramuscular artesunate as definitive treatment for severe malaria at lower-level facilities, especially when access to referral facilities is challenging. The study was to provide insight as a formative step into the conduct of future experimental studies to ascertain the feasibility of the use of intramuscular artesunate for definitive treatment of severe malaria in lower level facilities where access to referral care is limited.

**Methods:**

This qualitative study was done across three southern States in Nigeria (Oyo, Cross River and Enugu). Key informant interviews were conducted over a period of three months between October and December 2014 among 90 purposively selected health workers with different roles in malaria case management from primary care to policy level. A thematic content analysis was used to analyse data.

**Results:**

Overall, most of health workers and other key informant groups thought that the use of intramuscular artesunate for definitive treatment of severe malaria at lower-level facilities was possible. They however reported human resource and infrastructure constraints as factors affecting the feasibility of intramuscular artesunate use as definitive treatment for severe malaria in lower-level facilities.. Specifically identified barriers included limited numbers of skilled health workers available to manage potential complications of severe malaria and poorly equipped facilities for supportive treatment. Intramuscular artesunate was considered easy to administer and the proximity of lower-level facilities to communities was deemed important in considering the possibility of its use at lower-level facilities. Health workers also emphasised the important role of operational research to provide additional evidence to guide the implementation of existing policy recommendations and inform future policy revisions.

**Conclusions:**

From the perspective of health workers, use of intramuscular artesunate for definitive treatment of severe malaria at lower-level health facilities in Nigeria is possible but dependent on availability of skilled workers, well-equipped lower-level facilities to provide supportive treatment There is need for further operational research to establish feasibility and guide the implementation of such an intervention.

**Electronic supplementary material:**

The online version of this article (doi:10.1186/s12913-016-1811-x) contains supplementary material, which is available to authorized users.

## Background

Morbidity and mortality due to severe malaria remains disproportionately high in sub-Saharan Africa, with about 90 % of the world’s severe and fatal cases affecting young children in this region [1]. Despite modest improvements in the last decade, malaria still remains a major public health problem in Nigeria. In 2013 alone, about 7.8 million confirmed malaria cases 6000 malaria deaths were reported in Nigeria [2]. With a population of approximately 172 million, the country continues to report more deaths due to malaria than any other country in the world [2]. As delay in receiving effective treatment is a major driver of this high malaria mortality [3], improved access to prompt and appropriate treatment is critical to reducing deaths attributable to malaria. A review of hospital records in tertiary facilities in western Nigeria indicate that 11.3 % of hospital admissions were due to severe malaria with 89 % being children less than 5 years of age [4].

In Nigeria, three different health care levels exist with each having a well-defined role in the continuum of care for malaria cases in the communities served. These include primary (lower), secondary and tertiary (referral) care levels respectively. Primary care facilities (lower-level facilities) are the entry points for formal health service uptake and are often most accessible to the communities. The clinical cadres of personnel commonly found at the primary care level include junior and senior community health extension workers (CHEWs); community health officers (CHOs); Nurses/Midwives; public health nurses and medical doctors. CHEWs are cadre of health workers with two-year basic training on community mobilization, management of common ailments and pre-referral treatments. CHOs are experienced CHEWs or Nurses with additional one-year management training on primary healthcare delivery. The number and cadre available at any given primary health facility vary and depend on the category of primary facility, which include health post (HPs)-expected to have at least one CHEW; primary health clinic (PHCs) offering largely out-patient services with at least six CHEWs. Comprehensive health centres (CHCs) are the third category with nine CHEWs, one CHO, one public health nurse, three nurses/midwives and a medical doctor. The CHC in most cases have in-patient and maternity services. The anticipated service benchmarks, minimum infrastructure and human resource requirements stated above (are set by the national primary care minimum service package [5]. However, previous health facility assessments at this primary level of care have shown wide variations in service delivery context in relation to these set benchmarks [6–8]. The primary health care level provides pre-referral treatment for severe malaria cases [4, 9] and also extends into the community with CHEWs mandated to spend a proportion of their time in communities within the catchment area of the primary health care facilities. CHEWs also supervise a broad range of resident community health volunteers or community caregivers, who predominantly manage cases of uncomplicated malaria. Secondary and tertiary care facilities offer comprehensive health care and definitive management for both uncomplicated and severe malaria [9].

According to WHO handbook on severe malaria, severe *falciparum* malaria is defined as a patient with parasitological confirmed malaria who has one or more of the following clinical features: impaired consciousness; prostration; more than two convulsive episodes within 24 h; metabolic acidosis; acute pulmonary oedema and acute respiratory distress syndrome; circulatory collapse or shock (systolic blood pressure <80 mm Hg in adults & < 50 mmHg in children); severe anaemia (Hb <5gm/dl or haematocrit <15), renal impairment; jaundice and abnormal bleeding. the Nigerian national malaria treatment guidelines for pre-referral treatment options for severe malaria in children, in order of preference are: intramuscular artesunate (IM AS), rectal artesunate or intramuscular quinine [9]. Intravenous or intramuscular artesunate is used for definitive treatment of severe malaria at higher-level health facilities. It is administered as Intravenous/Intramuscular 2.4 mg/kg is given initially at time 0, then at 12 h and 24 h and then daily until the patient can tolerate oral medications with a full three-day course of Artemisinin-based combination therapy (ACTs) [9].

In resource-poor settings found in many parts of Nigeria, healthcare workers are faced with making potentially life-saving decisions related to the management of severe malaria. Some of these include making referrals in the presence of uncertainty of availability of timely referral services which could potentially further put the patient at risk. It is within this context that pre-referral treatment, which potentially could offer the patient more time in the presence of any delays in receiving appropriate definitive treatment for severe malaria, is recommended. However, even with such recommendations, health systems challenges exist that negatively impact on clinical outcomes. In these settings, referral practices are often sub-optimal [6–8, 10], hampered by poor transportation networks and long distances to referral centres. Consequently, transit time to referral facilities may be prolonged leading to delays in initiation of definitive treatment resulting in deaths [11].

In view of the constraints related to timely access to definitive and supportive care at referral facilities, definitive management of severe malaria in primary facility settings maybe necessary in the absence of accessible referral to secondary and tertiary facilities. The provision of definitive treatment at lower-level health facilities could further shorten potential waiting times and reduce the occurrence of incomplete treatment resulting from failure to access definitive treatment after initial pre-referral administration of artesunate, especially within settings with poor access to proper referral facilities.

Definitive treatment of severe malaria with IM AS at lower-level facilities within this context may present a life-saving option to be considered given its efficacy, ease of administration and excellent safety profile [12, 13]. Deployment of such an intervention needs to be informed by contextual evidence of where and when the intervention is most appropriate. There is currently limited information on stakeholder perspectives and views on the use of IM AS in resource-limited settings. Exploring health worker perspectives on the possibility of IM AS use at lower-level facilities is an important step in determining if and where this approach could be considered as an option. The objective of this study was therefore to assess health workers’ perspectives on the possible use of IM AS for definitive treatment of severe malaria in lower-level health facilities.

## Methods

### Study sites

This qualitative study was conducted in three southern States in Nigeria (Oyo, Cross River and Enugu) as part of the Improving Severe Malaria Outcomes (ISMO) project which has an overall goal to reduce mortality from severe malaria in Ethiopia, Nigeria and Uganda. The states were purposively selected to represent areas of the country with malaria control intervention focused at strengthening the management of severe malaria.

### Sample and participants

Study participants were drawn from key influencers who held roles in the management of severe malaria. They were identified through a preliminary stakeholder analysis and include: state-level policy makers, academia, development and implementing partners’ technical staff, facility-based health workers and community caregivers. Policy makers included management and programme staff at the Ministry of Health and hospital management boards. Academia comprised of academic staff in teaching hospitals and other medical training institutions. Facility-based health workers included medical staff at the primary, secondary and tertiary health facility levels.

The sampling procedures are as stated below:

- Policy makers: 24 (eight in each state) key department/unit heads were purposively selected for interview in the Ministry of Health (MOH) and hospital management board (HMB), based on their involvement in malaria interventions in the three states.
- Academia: Six academics, one each from all of the teaching hospitals in the three states were purposively selected based on their research interest in malaria.
- Malaria implementation partners: Nine technical staff in each of malaria control implementing partners in the three states were purposively selected.
- Secondary health facilities selection: A random sample of 6 facilities (2 from each of the 3 States) were selected from a sample frame of thirty-six (36) facilities with evidence of prior training of health workers on the definitive management of severe malaria, according to national malaria treatment guidelines. The sample frame was compiled from training database provided by the three state ministries of health for trainings done within 6 months prior to the interview.
- Primary health facilities selection: A multi-stage sampling process was used for selection of health workers from primary-level health facilities. Stage 1 involved random selection of 1 local Government Area (LGA) from sample of all LGAs in each State. Stage 2: List of all primary facilities in each selected LGA with staff trained on the treatment of malaria within a period of 6 months prior to the interview was compiled as sampling frame, 1 facility was randomly selected in the selected LGA, bringing the total to 3 primary health facilities sampled.
- Community caregivers selection: All the community caregivers under the supervision of the selected LGA were selected for interview. Each primary facility supervised a minimum of 6 caregivers.

In each selected facility, all consenting clinical workers on duty were selected for interview until all available respondents at the time of the visit were interviewed. Interviews in each health facility were conducted over a maximum of two days depending on the availability and willingness of the clinical staff to take part in the study. Teams of interviewers moved from one facility or office to another to conduct interviews, thus, each team covered a given number of health facilities. Half of the participant were facility-based health workers and the remainder were policy makers, academicians and community-based caregivers as outlined in Table 1.

**Table 1:** Profile of key informant

Category	Gender		Average age (Years)	Experience in current position (Av. Years)	Highest educational qualification				Number of Participants (*N* = 90)				
	Male	Female			Post-graduate	Graduate/professional qualification	Secondary/High School	Primary/Basic school	Cadre	Cross River	Enugu	Oyo	Total
Policy Makers	12	6	50.44	3	15	3	0	0	Director	3	6	6	15
									Manager	1	1	1	3
Academia	3	3	44.83	12	3	0	0	0	Medical Doctor	1	1	1	3
									Nurse	2	0	0	2
									Pharmacist	1	0	0	1
Partners	3	6	42.33	2	6	0	0	0	Medical Doctor	2	2	5	9
Secondary Facility Workers	11	15	41.31	7	3	23	0	0	Medical Doctor	4	2	5	11
									Nurse	2	3	3	8
									Pharmacist	1	3	3	7
Primary Facility Workers	5	14	43.32	12	0	19	0	0	Medical Doctor	2	0	0	2
									Nurse	5	1	3	9
									CHEW	3	4	1	8
Community Caregivers	1	11	43.33	3	0	1	7	4	Volunteers	0	5	7	12
													90

Prior to interviews, meetings were held with officials of the malaria control unit of the state Ministry of Health in the three states to provide more information and to seek approval to conduct the study and agree on modalities for the study. Heads of the selected facilities were contacted on phone to give notice, secure appointments for interviews and agree on convenient visiting period, bearing in mind the work load and schedules at the facilities. Participants in the academic institutions were contacted for interview appointments in their individual capacities.

### Data collection

Data was collected by trained research assistants in each state, using a key informant interview guide that was developed and pre-tested by the research team. Research assistants were trained for a period of three days on the study protocol, interviewing skills and note-taking. Training events conducted included hands-on sessions, practical demonstration exercises and role plays. A pre-test was conducted on the data collection tools in Enugu state. The key informant interview guide was pre-tested among twelve health workers across the categories of the targeted respondents to ensure that questions were non-ambiguous and collected the data required to answer the research questions. Following the pre-test, no significant changes were made to the tools as it was found suitable for collecting the data. The pre-test interviews were not included in the final sample of the interviews conducted. The interviews explored knowledge and views about IM AS use for management of severe malaria at lower-level facilities; perceived benefits and barriers and strategies to address the barriers. Interviews were conducted in English in a quiet and private environment, devoid of distractions.

Data was collected over a period of three months between October and December 2014. Interviews were conducted in English in a quiet and private environment, devoid of distractions. The interview guide predominantly had open-ended questions and the average duration of each interview was 30 min. During the data collection, the research assistants worked in pairs consisting of an interviewer and a note-taker. Interviews were audio-recorded to ensure no information was missed. Daily team meetings were held to discuss field experiences and review daily activities and quality of work with remedial actions taken where necessary.

### Data processing and analysis

Audio recordings from interviews were transcribed verbatim by the research assistants and supplemented with notes taken during the interview. The interviews were reviewed against audio recordings by the research coordinator to ensure consistency, completeness and quality. This information was typed and saved electronically in password-protected files. A thematic content analysis was conducted and data was coded and summarised into emerging themes based on the questions asked, by creating a data table from each transcript. A four-member study team conducted the coding exercise; similar themes were categorized together based on the frequency of recurrence and connection. Data that was less frequent, unique and divergent, was also scrutinized. The study team held discussions to gain more comprehension of the emerging themes and to draw comparisons on the most important themes. A code book was developed to outline the themes and sub-themes. Verbatim quotes were used to illustrate the themes and sub-themes (Additional file 1).

### Ethical considerations

Ethical approval for this study was obtained from the National Health Research Ethics committee of the Federal Ministry of Health, Abuja (Protocol approval number NHREC/01/01/2007-01/12/2014). Permission to conduct the study was also obtained from the malaria control authorities in each of the three participating States. Participation in the study was voluntary and written informed consent was obtained from all the participants. Permission for audio-recording interviews was also obtained at the start of each interview. Identifying information as not recorded for any of the participants. Instead, pseudonyms were used to protect the participants’ identity and to ensure that the data could not be linked back to them.

## Results

From the narratives, participants identified a number of perceived benefits and challenges related to the introduction of IM AS for definitive treatment of severe malaria at lower-level health care levels. Broadly, there were four main themes: human resources barriers; infrastructural suitability; drug-related profile of IMAS as enabling factor for its use at lower-level facilities and role of evidence in driving implementation of policy and policy revision.

### Theme 1: Human resource barriers to the implementation of IM AS at lower-level facilities

A number of human resource issues were commonly cited as major factors affecting the possible use of IM AS use at lower-level facilities and this was a key theme in most of the narratives. Specifically these issues included the inadequate numbers and uneven distribution of health workers, lack of skilled health workers at lower levels to manage severe malaria complications and the potential risk of drug misuse for uncomplicated malaria leading to resistance.

#### Sub-theme 1.1: Non-availability of health workers as a barrier to IM AS roll-out at lower-level facilities

The issue of non-availability of skilled health workers at lower-level health facilities was highlighted as a major barrier to the use of IM AS at this level. For many of the participants, availability of skilled health workers was a major determinant for the use of IM AS at lower-level facilities.

*“Most of the lower health facilities in Nigeria are not properly staffed. Cases whereby unqualified or semi-qualified staff are found, you cannot leave them to administer such injection.” Facility-based health worker from Cross river state*

Participants felt strongly that the cadres of health workers in most of the lower-level health facilities, who in most cases are CHEWs, were not sufficiently knowledgeable and skilled to manage severe malaria. CHEWs undergo a two-year basic training in management of common ailments using a “standing order” (Ministry of Health) staff standard operating procedures).

*“Cadre of personnel at these lower-level health facilities are the CHEWs, in most cases who do not have the comprehensive medical knowledge on management of patients, their capacity is low, they are not skilled to know how to constitute and give correct dosage.”* Policy maker from Oyo State

Other participants noted the ability to manage severe malaria was not necessarily about the facility level, but the skills of the clinical workers.

*“What determines whether they can treat is the health worker. Any facility that has a doctor or a trained nurse can carry on with the treatment”.* Facility-based health worker from Enugu State

#### Sub-theme 1.2: Task shifting as a response to health worker maldistribution

Some participants acknowledged the uneven distribution of health workers in the country, with highly skilled staff preferring to work in the urban areas, while some of the health facilities in rural areas had unskilled staff. Different audience groups suggested that if trained health workers available at lower-level facilities, they could treat severe malaria using IM AS if other conditions required for good clinical management are met. To those with this opinion, particularly the policy makers, it would serve as a form of task-shifting to respond to the human resource for health challenges at this level:

*“It is a form of task-shifting because there is concentration of well trained staff in the cities and incidentally most of these severe malaria cases occur in those hinterland, so there is a need to task-shift and train those people who are in the hinterland with lower-level healthcare facilities to be able to manage severe cases of malaria.”* – Policy maker from Enugu State

Other participants highlighted that training cadres of health workers from lower-level health facilities to manage severe malaria would be life-saving for rural communities without access to highly qualified health workers.

*“There are so many rural communities that do not have hospitals; you don’t expect someone who is ill there to wait until he comes to city before he gets Injection. If they [health workers] there are taught how to use IM AS and how to look out for likely complications, it will be beneficial to mankind.”* Policy maker from Enugu State

Most participants at facility level suggested that with training the health workers would be able to manage severe malaria using IM AS. In particular, participants at lower-level facilities (PHC) felt confident that if they are empowered in appropriate case management practices, they would effectively take on this role. PHC workers stated:

*“Give us training and we will have the knowledge. Provide us with the admission facility like beds, because the policy said before now, refer when you see such cases, but when the policy changes and we are trained, we will able to handle it.”* Lower-level health worker in Oyo State

Participants stated that a shift in policy allowing first level health workers to teat severe malaria would empower them to start treating severe malaria by providing the tools and competencies to do so:

*“The fundamental reason for referring severe malaria cases is because we have not been asked to start treating the cases. Because even where there is doctor, when the doctor writes the drugs, it is the nurse that will give the drug. So if they ask us to start treating the sickness, we would start treating severe malaria cases.”* – Lower-level health worker from Oyo State

There were suggestions that updating the national malaria treatment guidelines with clearly defined case definitions of severe malaria would provide a useful guide for health workers, making it possible for them to use IM AS at lower care levels. Other participants suggested that severe malaria cases in the absence of serious complications could receive definitive treatment at lower-level facilities, while those with serious complications should receive pre-referral treatment before referral to higher levels of care.

*“They can do it since they are allowed to give IM injections but the level of severe malaria must be graded to indicate the stage they can be allowed to handle.”* Policy maker from Cross River State

#### Sub-theme 1.3: Inability to manage complications associated with severe malaria

In stark contrast to the proponents of task-shifting in this study, there were also strongly divergent views particularly among policy makers who did not support definitive treatment of severe malaria at lower-level facilities. There was concern particularly among policy makers that allowing the roll-out of IM AS at lower-level facilities would result in cadres who are not sufficiently skilled attempting to manage severe malaria cases beyond their ability and failing to refer resulting in increased death.

“*They will over-step their boundaries (in treating other severe cases) and will think they are the same as doctors and will make them not to refer cases, thereby making people to die.”* - Policy maker from Oyo State.

Additionally, there were concerns that use of IM AS was not simply a matter of administering injection but being able to manage resultant complications.

*“It is all conditional, it is not just giving injections but the staff must be able to manage other complications of malaria. If the staff is properly trained on these areas and knows the best time to refer – okay [it’s okay by me]* …*”* – Policy maker from Oyo State

The participants retreated the concern that insufficiently skilled cadres at lower-level healthcare facilities would not be in a position to holistically manage the complications associated with severe malaria*.*

*“If you are treating severe malaria, you are not looking at injectable AS alone. If a person has severe malaria, we also have other complications that need to be treated which they may not be able to treat at these lower health facilities.”* – Academic from Cross River State

*“They should not be empowered at all because they cannot manage other complications that come with severe malaria but they can give pre-referral treatment and not the total management of severe malaria.” –* Policy maker from Oyo State

### Theme 2: Infrastructural suitability for the use of IM AS at lower-level facilities

Participants highlighted potential challenges with other requirements for good and comprehensive service delivery such as the non-availability of appropriate equipment including admission beds, drug storage facilities, laboratories, oxygen and blood transfusion equipment. As a facility-based health worker observed:

“*The problem is that of the drug, lack of storage facilities and lack of adequate equipment.”*

While the lack of infrastructure was a concern for some of the participants, close proximity of the lower-level health facilities to communities was perceived as a benefit of using IM AS at lower-level health facilities. Most participants stated that lower-level facilities were closer in proximity to the general population making it easier for patients with severe malaria to access timely care, especially where there are weak or non-existent services.

*“Ït would be nice to have lower-level facilities treat severe malaria cases because they are nearer to the people. Community members prefer to go to the primary health centre and also it reduces the transportation fare”* - academic from Enugu State

### Theme 3: Drug-related profile of IM AS

Some participants stated that the non-availability and inconsistent supply of IM AS due to the high cost, lack of storage facilities and expiry of drugs before use, were potential barriers to the use of IM AS at lower-level facilities.

*Availability of drug, storage-so many, there is no electricity to maintain it at the right temperature especially in remote location, since there is less incident of severe malaria, the possibility of the drug expiring before use”*- A policy maker from Cross River State.

Some health workers at the facility level across the three States also expressed the fear that there would be risk of IM AS misuse by health workers at this lower-level for treating uncomplicated malaria.

*“There is the possibility of using IM AS for any malaria case which may not even be severe malaria”-* A higher-level health facility worker from Enugu

Other health workers who highlighted the same concern that misuse would lead to drug resistance *“There may be abuse. It may not be used for severe malaria alone and may lead to drug resistance”* – An academic from Teaching hospital in Oyo State

Nonetheless, most participants agreed that IM AS has some positive attributes that make it suitable to be administered at lower-level health facilities. Accordingly, the majority noted that IM AS is easy to administer and requires less time and medical devices for administration. They also noted that IM AS administration involve less procedure than IV AS, so it can be done by nurses when doctors are not available.

“*IM AS has easy administration because a nurse can easily give an IM instead of waiting for the doctor to come and give IV –* A technical-lead with a partner in Oyo State

Another perceived benefit across all categories of respondents was that IM AS may be best suited for some patients such as patients with collapsed veins:

*“Is easy to administer, saves time and less skilled personnel can use it as it helps to avoid looking for vein in people who are malnourished.”* – A higher-level facility worker in Cross River State

Other responses suggested that IM AS is slower than intravenous artesunate in action, hence side effects can easily and quickly be reversed: According to a policy maker:

*“The fact that IM AS is slower in reaction is a benefit because side effect can easily and quickly be reverse unlike IV that goes faster into the blood stream.”* – A policy maker in Enugu State

### Theme 4: Support for evidence-based review of polices and implementation of guidelines

The majority of participants supported periodic policy reviews to expand the use of IM AS to definitive treatment of severe malaria at lower-level facilities. However they noted that implementation of such a policy change would only be successful if operational research and pilot studies informs on the operational feasibility of proposed modifications to this new approach and suggested changes are made in a consultative manner, taking on board practical experience and understanding of the context. Policy makers stated:

*“There is a need to have a roundtable discussion, do some operational research and have some findings. If the evidence based studies carried out in various areas come out with various advantages on the treatment of severe malaria, they should be included.”* – A policy maker from Cross River State

## Discussion

The study findings indicate that the health workers and other respondents felt that definitive treatment with IM AS in some lower-level facilities may be possible as an approach to improve treatment outcomes of patients with severe malaria. The proximity of lower-level facilities and relative ease of access and of administration of IM AS were stated as making it a potential option to be explored for use at lower-level health facilities. However, the heath workers noted that introduction of IM AS at lower-level could be hindered by inadequate and unskilled health workers, lack of equipped facilities, inconsistent drug supply and lack of knowledge among health workers and other supportive measures required for the management of concomitant serious complications resulting from severe malaria at lower-level facilities.

Availability of skilled health workers at these lower-level facilities with the capacity to manage complications of severe malaria was considered to be crucial for the effective use of IM AS and to be able to achieve any anticipated improvements in treatment outcome at lower levels. At the primary level of care, the difference in scope and quality of services offered, present both an opportunity and a threat to effective management of severe malaria, the current status of these lower levels of care in terms of staffing could be a hindrance to such an approach [5, 6, 8, 10]. Indeed, recent assessments conducted by National Primary Health Care Development Agency in Nigeria (NPHCDA) have revealed that these lower-level health facilities were grossly understaffed and particularly lacked skilled personnel [6, 7]. Our study begins to highlight some critical issues for consideration in resource-poor settings that may be important such as the ability of the health workers at these lower levels to correctly recognise and manage severe malaria patients, which become more relevant in setting with limited access to referral health services.

These findings also suggest that additional training of health care workers at these lower-level facilities prior to implementation of IM AS to improve their technical capacity [10, 14] may be needed. Health workers identified potential skills gaps to include skills for appropriate diagnosis and recognition of severe malaria and knowledge and skills required for the management of complications associated with severe malaria. These challenges may be overcome by health worker training programmes, which have been shown to improve case management and treatment outcomes [15, 16]. These programmes could be tailored to include the needs of different cadre of health workers.

This study found that respondents thought the ease of IM AS administration and convenience for patients in circumstances where venous access is challenging made its use for definitive treatment of severe malaria at the lower levels amenable. This finding is consistent with a recent study in DR Congo, where health care providers perceived the handling and administration of injectable AS to be easy [17]. In addition, evidence of the non-inferiority of simplified dosing regimens of IM AS with once daily dosing for 3 days [13] provide much needed assurance towards promoting IM administration of AS. The concern however raised by health workers in the study about the risk of drug misuse leading to resistance is pertinent threat and while there is no documented evidence to suggest that this is yet a problem for IM AS, it is worth noting as a potential challenge.

The definitive treatment of severe malaria with IM AS at lower-level facilities was considered possible by respondents due to the close proximity of these health facilities to communities, thus opening up the possibility for more prompt access to treatment; however, challenges were cited with regards to the availability of requisite equipment for supportive care and treatment of severe malaria within lower-level facilities. Health facility infrastructural capacity, availability and adequacy of essential laboratory services and medicines, other supplies to support appropriate diagnosis and management of severe malaria are also vital [10, 14]. Deliberate efforts are required to make some designated lower-level facilities function optimally to be able to provide the required service, especially in areas with poor access to referral facilities, this would increase timely access to parenteral treatment, thus contributing to reduction in malaria related mortality. The anticipated improved access to IM AS, as suggested, closely mirrors the concept and approach in improving access to integrated community case management (iCCM) that has been shown to increase treatment coverage, improve timeliness of treatment and reduce mortality [18]. Such innovative interventions are critical in high malaria burden settings where malaria contributes significantly to mortality especially among children less than 5 years of age.

The study findings indicate that further research is needed to inform policy reviews and the practicalities of implementation at scale at lower-level facilities. This is crucial as changing malaria policy and translating policies to practice are generally complex and long processes [19, 20]. The important role of evidence in supporting this process is also highlighted in other national settings [21]. In the context of this study, the perceived acceptability by the health workers is a good starting point as it reflects significant support that could minimise delays in translation of accrued evidence into appropriate policies changes.

### Strength and limitations of the study

The main strength of this study is the qualitative mode of inquiry used and the diversity of stakeholders purposively interviewed ranging from frontline health workers, academics and policy makers. This ensured that the various perspectives were taken into account from the information-rich participants. Some identified limitations of this study include a recognition that the study only aimed to capture stakeholder perception and opinion on the possibility rather than true feasibility in real life situations in lower-level facilities, as this could be different from views expressed. It will therefore be important to assess feasibility in an experimental study with a safety component within the context of a pilot intervention. This study only considered the health worker perspectives and not the end user perspectives on the acceptability and feasibility of IM AS use at lower level facilities, therefore it cannot be construed that their perspectives on acceptability would be similar.

Whereas the study findings cannot be generalised, they highlight the important contextual issues on the possibility of using IM AS at lower-level facilities in Nigeria and may need to be considered in similar service delivery settings. This evidence is useful for the National Malaria Elimination Programme and its partners in Nigeria to initiate discussions on potential next steps to operationalise the use of IM AS for definitive treatment of severe malaria at lower-level facilities which often serve as the first and only point of care for many communities.

## Conclusion

The findings indicate that use of IM AS for definitive treatment for severe malaria at Lower-level health facilities in Nigeria is possible. However, the ability to implement this was premised upon certain conditions being in place. These conditions include the availability of adequate skilled health workers, equipment and supplies for supportive treatment of severe malaria at lower-level facilities. Specifically, skilled health workers, refresher training for health workers on use of IM AS for management of severe malaria and associated complications or co-infections and improvements in infrastructure for supportive treatment will be crucial for improving the feasibility of IM artesunate use at lower-level health facilities. Further operational research is however needed to better inform optimal implementation approaches in this setting.

## Additional file

Additional file 1: Qualitative Coding Scheme for Key Informant Interviews. (DOCX 15 kb)

## Abbreviations

ACTs: Artemisinin-based combination therapies
AS: Artesunate
CHEWs: Community health extension workers
CHOs: Community health officers
iCCM: Integrated community case management
IM AS: Intramuscular artesunate
ISMO: Improving Severe Malaria Outcomes
MOs: Medical officers
NPHCDA: National Primary Health Care Development Agency in Nigeria
PHC: Primary Health Care
