# Supplementary material for: Health worker perspectives on the possible use of intramuscular artesunate for the treatment of severe malaria at lower-level health facilities in settings with poor access to referral facilities in Nigeria: a qualitative study
Source: BMC Health Serv Res. 2016 Oct 12;16:566. doi: 10.1186/s12913-016-1811-x (PMC5059903; doi:10.1186/s12913-016-1811-x)
Supplement: Additional file 1: — Qualitative Coding Scheme for Key Informant Interviews. (DOCX 15 kb) [file 12913_2016_1811_MOESM1_ESM.docx]

# Additional file 1: Qualitative Coding scheme

| **Main themes** | **Codes** | **Exemplar quotes** |
| --- | --- | --- |
| Facility factors  (Question 4) | 1.1 Accessibility to the population  1.2 No referral facilities  1.3 Lack of equipment  1.4 Provision of admission facilities | “It would be nice because they are nearer to the people. Community members prefer to go to the primary health centre and also it reduces the transportation fare.” (Ac)  “The problem is that of the[…] lack of storage facilities and lack of adequate equipment.” (HWS) |
| Personnel factors  (Question 4) | 2.1 Availability of skill health worker  2.2 Task-shifting  2.3 Lack of training on IM AS use  2.4 Lack of capacity to manage complication  2.5 Lack of capacity to manage co-infection  2.6 Misuse of IM AS for uncomplicated malaria  2.7 Inadequate skilled health workers  2.8 Manage severe cases beyond ability | “Give us training and we will have the knowledge. Provide us with the admission facility like beds, because the policy said before now, refer when you see such cases but when the policy changes and we are trained, we will able to handle it.” (HWPHC)  “Cadre of personnel at these lower level health facilities are the CHEWs, in most cases who do not have the comprehensive medical knowledge on management of patients, their capacity is low, they are not skilled to know how to constitute and give correct dosage.” (P)  “They will over step their boundaries (in treating other severe cases) and will think they are the same as doctors and will make them not to refer cases thereby making people to die.” (P) |
| Drug-related factors  (Question 3) | 3.1 Ease of administration  3.2 Less time for treatment  3.3 Less expensive  3.4 Fewer technicalities  3.5 Slow action, reversible side effects  3.6 Suitable for patients with collapsed vein  3.7 Inconsistent supply of IM AS  3.8 Inconsistent supply of consumables  3.9 Drug expiry | “[…] Easy administration because a nurse can easily give an IM, instead of waiting for the doctor to come and give an IV.” (HWS)  “Is easy to administer, save time and less skilled personnel can use it as it helps to avoid looking for vein for people who are malnourished.” (HWS)  “The fact that IM AS is slower in reaction is a benefit because side effect can easily and quickly be reversed unlike IV that goes faster into the blood stream.” (P) |
| Policy review process  (Question 5) | 4.1 Further research for generation of additional evidence  4.2 Translation of policy into guidelines  4.3 Revise and disseminate policy and guidelines | “There is need to have a round table discussion, do some operational research and have some findings” (P)  “If the evidence based studies carried out in various areas come out with various advantages on the treatment of severe malaria, they should be included” (P) |

*P=*policy maker; *Ac= academia; HWPHC health worker at PHC; HWS =health worker at secondary level*
